# Clinical Cases and Provings

**Published:** 1888-10

**Authors:** E. W. Berridge

**Affiliations:** London


### CLINICAL CASES AND PROVINGS.

E. W. Berridge, M. D., London.

(1) *Phosphorus.*—April 22d, 1888.—Miss L. L., about forty-five years old. Six weeks ago when going into cold wind, felt something strike icy-cold in centre of lower chest. Since then, cough caused by lying on right side, aggravated by eating and sometimes for a short time afterward; also worse on rising from bed in the morning; sputa thick, yellow, tasteless; cough causes pain like a smarting at front of throat, about root of tongue; constant pain in centre of lower chest as if stopped up; cannot draw a long breath on account of a feeling of weight in centre of lower chest, relieved by sighing.

I selected *Phosphorus* from Lee's *Cough Repertory*, and gave one dose of Cm (F. C.) at seven P. M.

April 23d.—Last night cough was worse when lying down in any position; no cough this morning; throat, chest, and breathing much better.

April 26th.—Has had no return of cough; chest feels much stronger.

April 29th.—Quite well, and has remained so.

(2) *Conium.*—August 21st, 1886, an American patient, recommended to me by the lamented Dr. Pearson, wrote as follows: "A member of my household, a young woman of twenty-five, has been for some months annoyed by red spots of different sizes, averaging perhaps half an inch in diameter, which appear on her legs, and sometimes on the right side of abdomen. They are not raised, and there is no itching or burning; but they sometimes turn bluish or green and look like bruises, and at times her legs feel numb; she seems strong and otherwise well, except that her feet are tender and sometimes swell and burn when she stands a long time." On writing for further information as to constitutional history, my patient replied August 26th, as follows: "She has observed the spots for more than a year; they once disappeared under allopathy, but came back in a few months. Her feet are sometimes cold on first lying down at night, but are usually burning both day and night, often swelled and feel stiff. This tenderness and swelling has always troubled her since she grew up and began to work. Her mother has suffered much in this way, and often has erysipelas in the legs after exposure to cold and over-fatigue. The veins in patient's feet, forearms, hands, and under knees, are much swelled; the swollen veins throb, but are not otherwise painful; on pricking the finger the blood does not flow very quickly and is quite thick; menses have always been regular until since coming to Europe; since then irregular, and usually some days too soon; she always has had pain the first day caused by clots, but after that has been free from pain; menses last four or five days; her flesh bruises easily. Her father died of phthisis. Until these symptoms appeared, she has been very strong and well." On August 28th I sent *Conium*$^{10m}$ (Fincke), a dose in water twice daily for fourteen days.

September 17th I received the following report: "Decided improvement. The spots are more in number, but much smaller, and show a tendency to move, appearing and disappearing. On the whole they are lower down on the legs. The swelling and burning of feet is also less troublesome (the weather has been cooler). The small veins which were so deep a purple are much lighter in color and less conspicuous." *No medicine.*

October 6th reports, "Much better. The spots on the skin have entirely disappeared. The last menses were more comfortable, less copious, and no pain except on second day, when she thinks she took cold from a sudden change in weather. The only remaining trouble seems to be a feeling of languor and disinclination to exert herself, not natural to a person of her energetic temperament." *No medicine.*

November 7th, reports, "The spots on the skin entirely disappeared some time since and have not come back. Caught cold at last menses, which in consequence only lasted two days, with considerable pain, and followed by a troublesome leucorrhœa, which is now better. Otherwise she seems very well." This patient I treated entirely by correspondence without ever having seen her. I received no further report.

(3) *Calcarea carbonica.*—Major —— wrote as follows: "John C., groom, aged thirty-six, got a chill and cold about December 7th, 1883, and ever since is deaf in left ear; sound in left ear as of a lot of trees waving; no pain. Aggravated by driving; feeling as if a slide was let down over the ear, and sometimes it goes up, but quite deaf under any circumstances. With finger in right ear is stone deaf, could not hear a cart going by. Above is present state and condition of my groom. Can you do anything for him?"

On January 12th, 1884, I sent him one dose of *Calcarea carb.*$^{cm}$ (F. C.)

1885, February.—Major —— reported that it had cured his groom in a fortnight, and there had been no return.

(4) *Arnica.*—Mrs. C., aged fifty-four, consulted me June 11th, 1886. The left upper eyelid was ecchymosed, and also round left internal canthus. It came on suddenly two days ago, after rubbing it with a towel when washing. Has used *Arnica* lotion, but it has got worse. I ordered bathing with hot water, and gave *Arnica*$^{mm}$ (Fincke) in water twice daily.

June 19th, her husband reported that the eye improved in twenty-six hours, and that the discoloration had now nearly disappeared.

This case shows the superior action of a potency administered internally to the crude drug applied externally. Bœnninghausen pointed out years ago that even local injuries were often best treated in this way, and I have verified this in the case of sprains and bruises.

(5) *Arnica.*—October 31st, 1886, the same patient wrote to me. A week previously she had a severe fall down two-thirds of a spiral staircase, and as she is very stout and has a weak heart it was a serious matter. She was severely shaken, and her back and sides much bruised and very painful. She used Elliman's embrocation, which relieved her pain in back and right side, and the bruised appearance; but the pain at the heart increased. She now has pain in the muscles on left side of spine and around heart; every few minutes she draws a long breath which causes a very acute sharp pain at apex of heart, and it is getting worse. On November 1st I sent her three powders of *Arnica*$^{mm}$ (Fincke), a dose in water to be taken thrice daily for six days.

November 9th, reports improvement in twenty-four hours; now the bruised pain is almost gone, and only a little pain at heart, but riding in a carriage has increased it again. She has less frequent occasion to take a deep breath, and when she does so the pain is less acute. *No medicine.*

December 24th, writes to say that she has been quite free from pain for some time.

(6) *Sepia.*—Mrs. B., aged thirty-two, wrote July 23d, 1885, that for forty-eight hours she had suffered from irritation in vagina (she used to suffer from it during pregnancy, but she was confined about two months ago), worse during night and from friction; the irritation is a smarting and itching, and the vagina protrudes and is somewhat swollen, with a sense of weight there; the irritation prevents sleep. *Sepia*$^{cm}$ (F. C.) twice daily for fourteen days.

August 14th, wrote that the irritation and other symptoms ceased in four days and did not return. I have no doubt a single dose would have cured equally well. Some cases require a repetition, but I can now see that I have often unnecessarily repeated the dose in former years.

(7) *Causticum.*—May 1st, 1885.—Mrs. D. had sudden pains at four P. M. in inner side of right thigh where it joins the body as if a bruise were pressed on; worse when throwing left foot forward in walking and so bearing all the weight on right foot. At half-past eight P. M., the pain having continued, I gave her one dose of *Causticum* $^{cm}$ (Swan). In two hours she was better, and next day only a little pain was felt. On May 27th she reported that there had been no return.

(8) *Kali carbonicum.*—December 19th, 1884, Mrs. D. For four mornings at four or five A. M., violent, exhausting sneezing, lasting about two hours; during and after sneezing running of clear water from left eye and left nostril. *Kali carb.* $^{cm}$ (F. C.) one dose.

December 24th.—Had no return of symptoms till about seven P. M. December 20th, when the sneezing returned. On December 22d it returned about eight A. M., but much less severe. Last evening it returned slightly, and severely between four and five A. M. this morning, with running of left nostril and left eye. As the action of the dose appeared to have ceased, and the symptoms began to return in their original severity, I gave one dose of *Kali carb.* $^{3cm}$ (Fincke), which removed them at once and permanently.

(9) *Lachesis.*—July 23d, 1887, Mrs. —— complained that when walking on hard ground (not when ascending or descending stairs) a pain catches her in middle of left thigh, rather toward inner side, when she puts the left foot forward. There is a crack in bend of right thigh where it joins body; also cracks between second and third toes of right foot, and between all the toes except first and second of left foot. For the last ten days violent sweat on genitals, extending over lower abdomen nearly to umbilicus, with a feeling as if the skin there would come off if rubbed. *Lachesis* $^{mm}$ (Bœricke), one dose.

August 26th reports that the pain in thigh ceased at once. The cracks went away very soon. The sweat and sensation as if the skin would come off did not return. Now the cracks between toes have returned, also in bend of right thigh, with itching between left toes. *Lachesis* $^{9mm}$ (Fincke), one dose.

The symptoms speedily departed and have not returned to this day, July 31st, 1888.

(10) *Ignatia.*—April 27th, 1885.—Miss R. complained of pain like a bad bruise over right eye, worse on bending head down; the spot is very tender to pressure; exposure to wind brings on the pain; also she has pain like a tight band over vertex laterally. *Ignatia*$^{mm}$ (F. A.) twice daily for fourteen days.

July 4th.—Reports that the pain soon ceased. For a week the bruised pain over right eye has returned. The feeling of a tight band is felt occasionally on waking. A repetition of the medicine removed it, and when I last saw her, March 29th, 1886, it had not returned.

(11) *Alumina.*—Mrs. R., aged sixty-four. May 16th, 1885, complained that her stomach felt as if being scraped; the pain goes through to back; it is worse about two hours after food; it also comes on almost directly after stool. She has had it at times for nine or ten months. *Alumina*$^{cm}$ (F. C.) twice daily for fourteen days.

August 6th.—Reports that the symptoms ceased in about a week, and did not return till a week ago; it is now worse about two hours after food. *Alumina*$^{cm}$ (F. C.) twice daily for fourteen days.

August 15th.—Reports the pain just as severe, and accompanied by a pricking as if stomach and abdomen were being tapped with the bristles of a hair-brush. She had this new sensation last August, but less severely. Yesterday the pain was worse than ever. This was clearly an aggravation from unnecessary repetition of the dose, so I stopped it at once.

August 25th.—Reports much better; "never had medicine which acted so quickly" as leaving off the powders. The symptoms soon ceased, and when I last saw her, January 6th, 1886, there had been no return.

(12) *Lachesis.*—Mr. P., aged fifty-two. August 11th, 1886. Has had a bad cough for some time, treated allopathically in vain. Now has hacking cough all day with whitish, frothy sputa, formerly salty. Cough worse in damp weather; excited by tickling in throat; sputa on waking is harder, and a dirty yellow color. At times noisy escape of flatus downward when coughing. *Lachesis*$^{cm}$ (F. C.) twice daily for fourteen days.

August 25th.—Writes that cough is almost gone, very little accumulations during night; occasionally, between ten and eleven P. M., a little tickling high up in throat, causing cough for about ten minutes. Damp weather still increases cough. *No medicine.*

September 20th.—Reports cough quite gone.

(13) *Lycopodium.*—July 31st, 1886, Miss L. complained of an unpleasant taste in the mouth; all food tasted bitter; directly food or water touched the palate, it seemed to diffuse a bitter vapor through the mouth and throat. *Lycopod.*$^{cm}$ (F. C.) twice daily for fourteen days.

September 20th, reported that there was no change while taking the medicine, but that an improvement commenced directly afterward; the symptoms soon ceased, and did not return.

I have noticed the same phenomenon in several other cases, which is a strong argument against the repetition of the dose.

(14) *Kali carbonicum* $^{cm}$ (F. C.) removed a feeling of emptiness in chest after cough.

(15) *Thuja* $^{cm}$ (F. C.) removed by the following day, a feeling of upward pressure in soft palate, in a case of catarrh.

(16) *Carbo-veg.*—April 16th, 1886, Mrs. B. has been worried by her sister's illness in February. For last six weeks, headache on vertex, as if carrying an immense weight which would break through skull; she feels it all day, and whenever she wakes at night; it is worse from reading or writing, better by walking in open air. *Carbo-vegetabilis*$^{cm}$ (F. C.) twice daily for fourteen days. She slept better the following night. On 22d wrote, "I am getting on wonderfully." Soon cured.

(17) *Lachesis.*—August 6th, 1885, Miss R., aged twenty-nine, took *Lachesis* $^{cm}$ (F. C.) twice daily for fourteen days for a cough which it removed.

August 25th, reports a perfectly new symptom; for two last weeks, feeling of a hot iron at back of right eye.

(18) *Kali carb.*—October 18th, 1883, Miss B., aged sixty-four, complained of the following symptoms: Wakes *about three* A. M., with feeling of discomfort, and cannot get to sleep again, unless she first gets out of bed; then she passes much urine, which relieves her generally, and she can sleep; at the same time there is cramp in right elbow and shoulder. Since childhood, if she lies on right side, feels stagnant all over and cold, especially in feet; if she turns over on to left side, the blood feels as if it trickled back into feet, and she feels comfortably warm all over. If she goes to sleep on right side, she wakes with a start and feels cold. From 1878 to 1880, after worry and fretting about her mother's death, had retching *at three* A. M. *Kali carbonicum* $^{cm}$ (F. C.) every morning for seven days.

October 25th.—Wakes now after six A. M. instead of at three A. M., and without the usual discomfort; otherwise unchanged. Repeat medicine for seven days.

November 2d.—Sleeps naturally; no more cramp. *No medicine.*

November 9th.—Has been able to lie on right side, and one night slept on it without any discomfort; sleeps well. *No medicine.*

November 16th.—Weather has been colder, and she has not been able to lie so well on right side. *Kali carbonicum* $^{cm}$ (F. C.) twice daily in water for eight days.

November 26th.—Can lie on right side without any discomfort; sleeps well.

February 29th, 1884.—With the exception of a temporary difficulty in lying on right side last December, has had no return of symptoms.

(19) *Calcarea.*—The same patient complained that her feet were actually cold, and felt subjectively wet, as if she had damp stockings on. *Calc-carb.* $^{cm}$ (F. C.) removed this symptom. She was gouty, and her father had chalky gout.

(20) *Veratrum.*—January 20th, 1887, Mrs. B., aged thirty-five, complained of empty feeling in abdomen after stool, as if she needed food; also headache on vertex about five P. M. *Veratrum album* $^{cm}$ (Fincke) cured.

(21) *Causticum.*—Mr. A., aged forty-five, reports that about six years ago he took *Causticum* $^{6}$ three times daily, for deafness. It caused a feeling about apex of heart as if there was strings there breaking. Twice afterward he took the same medicine twice daily, and each time it produced the same symptoms, but to a less extent.

(22) *Rhododendron* $^{200}$ cured swelling and hardness of left testis, following gonorrhœa. He used to have headache before a thunderstorm.

(23) *Baryta carb.*—January 15th, 1888.—Mrs. B., aged thirty-seven, has been out of health for two or three weeks, from nursing sick children. Feels *weak at eight* P. M. Sleep bad, wants to lie in bed mornings. *Feels full after a mouthful.* Low spirited. *Baryta carb.* $^{cm}$ (F. C.) one dose at half-past eight P. M. Next day very much better, and soon cured.

(24) *China.*—A boy, aged fourteen; diarrhœa for nearly two weeks, during and after meals; to-day it has come on at half-past four A. M., quarter of six A. M., eight A. M., and three times since; not much in afternoon: stools dark, painless. *China* $^{cm}$ (F. C.) three doses cured.

(25) *Sulphur.*—Mrs. G. for four days (after convalescence from pneumonia) pain in both shins, especially left, worse when standing; outer side of left lower leg swelled; boring pain in left lower leg, like a gimlet going just below knee to ankle. *Sulphur* $^{dm}$ (F. C.) every four hours. Symptoms suddenly ceased in two days.

(26) *Mercur. vivus.*—Mrs. D. had pain in throat on empty swallowing, as if swallowing over a tender lump; first on right side, then also on left; throat inflamed. *Mercurius vivus*$^{cm}$ (F. C.) cured.

(27) *Rhus.*—Miss T., aged twenty, had pains like a knife in right abdomen, on walking. *Rhus tox.*$^{101m}$ (F. C.) cured.

(28) *Lycop.*—Miss T., suffering from phthisis, took *Lycopodium*$^{cm}$ (F. C.) alternate mornings for fourteen days. It caused feeling of a lump in left lower abdomen, with pain, making her feel lame when walking. Never had it before.

(29) *Rumex.*—For some weeks, when undressing at night, I had itching of legs below knees, removed only by violent scratching, sometimes so as to draw blood. One dose of *Rumex crispus*$^{cm}$ (Fincke) cured.

### Errata in Homœopathic Physician.

P. 428, line four, for *light* read *sight*.
P. 429, line fourteen, for *greenish* read *grayish*.
P. 366, line five from bottom, for *light* read *fight*.
P. 368, under *Thuja*, for *eructations* read *erections*.
P. 434, line four from bottom, for *Lact.* read *Lachesis*.

